# The Burden and Determinants of Non Communicable Diseases Risk Factors in Nepal: Findings from a Nationwide STEPS Survey

**DOI:** 10.1371/journal.pone.0134834

**Published:** 2015-08-05

**Authors:** Krishna Kumar Aryal, Suresh Mehata, Sushhama Neupane, Abhinav Vaidya, Meghnath Dhimal, Purushottam Dhakal, Sangeeta Rana, Chop Lal Bhusal, Guna Raj Lohani, Frank Herbert Paulin, Renu Madanlal Garg, Regina Guthold, Melanie Cowan, Leanne Margaret Riley, Khem Bahadur Karki

**Affiliations:** 1 Nepal Health Research Council (NHRC), Government of Nepal, Kathmandu, Nepal; 2 Nepal Health Sector Support Programme (NHSSP), Ministry of Health and Population, Government of Nepal, Kathmandu, Nepal; 3 Kathmandu Medical College, Kathmandu, Nepal; 4 Institute of Medicine, Tribhuvan University Teaching Hospital, Kathmandu, Nepal; 5 Ministry of Health and Population, Government of Nepal, Kathmandu, Nepal; 6 World Health Organization Country Office, Kathmandu, Nepal; 7 World Health Organization Regional Office for South East Asia, New Delhi, India; 8 World Health Organization Headquarter, Geneva, Switzerland; Medical University Innsbruck, AUSTRIA

## Abstract

**Background:**

World Health Organization (WHO) estimates for deaths attributed to Non Communicable Diseases (NCDs) in Nepal have risen from 51% in 2010 to 60% in 2014. This study assessed the distribution and determinants of NCD risk factors among the Nepalese adult population.

**Methods and Findings:**

A nationally representative cross-sectional survey was conducted from Jan to June 2013 on the prevalence of NCD risk factors using the WHO NCD STEPS instrument. A multistage cluster sampling method was used to randomly select the 4,200 respondents. The adjusted prevalence ratio (APR) was used to assess the determinants of NCD risk factors using a Poisson regression model. The prevalence of current smoking (last 30 days) was 19% (95%CI:16.6-20.6), and harmful alcohol consumption (≥60 g of pure alcohol for men and ≥40 g of pure alcohol for women on an average day) was 2% (95%CI:1.4-2.9). Almost all (99%, 95%CI:98.3-99.3) of the respondents consumed less than five servings of fruits and vegetables combined on an average day and 3% (95%CI:2.7-4.3) had low physical activity. Around 21% (95%CI:19.3-23.7) were overweight or obese (BMI≥25). The prevalence of raised blood pressure (SBP≥140 mm of Hg or DBP≥90 mm of Hg) and raised blood glucose (fasting blood glucose ≥126 mg/dl), including those on medication were 26% (95%CI:23.6-28.0) and 4% (95%CI:2.9-4.5) respectively. Almost one quarter of respondents, 23% (95%CI:20.5-24.9), had raised total cholesterol (total cholesterol ≥190 mg/dl or under current medication for raised cholesterol). he study revealed a lower prevalence of smoking among women than men (APR:0.30; 95%CI:0.25-0.36), and in those who had higher education levels compared to those with no formal education (APR:0.39; 95%CI:0.26-0.58). Harmful alcohol use was also lower in women than men (APR:0.26; 95%CI:0.14-0.48), and in Terai residents compared to hill residents (APR:0.16; 95%CI:0.07-0.36). Physical inactivity was lower among women than men (APR:0.55; 95%CI:0.38-0.80), however women were significantly more overweight and obese (APR:1.19; 95%CI:1.02-1.39). Being overweight or obese was significantly less prevalent in mountain residents than in hill residents (APR:0.41; 95%CI:0.21-0.80), and in rural compared to urban residents (APR:1.39; 95%CI:1.15-1.67). Lower prevalence of raised blood pressure was observed among women than men (APR:0.69; 95%CI: 0.60-0.80). Higher prevalence of raised blood glucose was observed among urban residents compared to rural residents (APR:2.05; 95%CI:1.29-3.25). A higher prevalence of raised total cholesterol was observed among the respondents having higher education levels compared to those respondents having no formal education (APR:1.76; 95%CI:1.35-2.28).

**Conclusion:**

The prevalence of low fruit and vegetable consumption, overweight and obesity, raised blood pressure and raised total cholesterol is markedly high among the Nepalese population, with variation by demographic and ecological factors and urbanization. Prevention, treatment and control of NCDs and their risk factors in Nepal is an emerging public health problem in the country, and targeted interventions with a multi-sectoral approach need to be urgently implemented.

## Introduction

The emerging pandemic of non-communicable diseases (NCDs) is creating major health challenges worldwide. Of the 56 million global deaths in 2012, 38 million (68%) were attributed to NCDs, with almost three quarters (74%) of these deaths occurring in low and middle income countries [1]. The World Health Organization (WHO) estimates that deaths attributed to NCDs in Nepal have risen from 51% in 2010 to 60% in 2014 [2,3]. A hospital-based study in Nepal estimated the prevalence of NCDs in non-specialist health institutions to be 31%, indicating that Nepal, like other developing nations, is facing the growing burden of NCDs [4]. These diseases are driven by many factors including ageing, rapid unplanned urbanisation and unhealthy lifestyles [5]. Although NCDs constitute a major public health problem in Nepal, how best to address NCDs at the primary health care level in Nepal is not understood [6].

Eight major risk factors (four behavioural and four biological) contribute most to the development of NCDs [7]. Tobacco use, harmful use of alcohol, unhealthy diets (high in salt, sugar and fat and low in fruits and vegetables) and physical inactivity are established modifiable behavioural risk factors for NCDs [7]. Among these, use of tobacco and alcohol is most common in Nepal [8]. Previous studies have reported a high prevalence of tobacco use in any form, especially among men (52% in males and 13% in females) [9]. Unhealthy diet is another major challenge in Nepal. High salt intake is more prominent among the rural population, who make up 83% of the total population of the country [9]. Low fruit and vegetable consumption is attributed to poverty, lack of purchasing power and increasing age [10]. In addition, although physical inactivity is not a major problem in the general population, it is clustered in urban and semi-urban populations [11], including in children and adolescents. This may be due to rapid and unplanned urbanisation, changes in lifestyle and globalization [12]. The 2007/08 national survey of NCD risk factors gave a national picture of behavioural and selected biological risk factors (overweight, obesity and raised blood pressure) using STEP I and II [8]. Periodic evidence including additional biological risk factors using all three steps was felt necessary to develop a national action plan for prevention and control of NCDs, and improve the design and implementation of preventive measures and public health interventions to reduce the burden of NCDs in Nepal. The need was further warranted as there are increasing reports of outpatient visits and inpatient admissions attributed to NCDs with 84% of total OPD visits and 90% of total inpatients discharged from hospitals accounting for NCDs as per the annual report of department of health services for the year 2011/12 [13].

This study assessed the epidemiologic distribution and determinants of risk factors for NCDs in the adult Nepalese population; specifically, the prevalence and determinants of behavioural and biological risk factors for selected NCDs in Nepal.

## Methodology

### Study design and sampling technique

A nationally representative cross-sectional survey was conducted from Jan to June 2013 on the prevalence of NCD risk factors using the WHO NCD STEPS instrument. A multistage cluster sampling method was used to select 4,200 respondents aged 15 to 69 years. Sample size was calculated using the prevalence of low fruits and vegetables intake (61.9%) from the 2007 STEPS survey [8] with an expected response rate of 80% as suggested in the STEPs survey guideline [14]. The primary sampling unit (PSU) of this survey was the Ilaka (an administrative unit at the sub-district level). Each Ilaka comprises of either a. 4 to 6 village development committees (VDCs) b. 4 to 8 wards of municipalities or c. 2 to 3 VDCs with 4 to 6 wards of municipalities combined together. Out of the 921 Ilakas in Nepal, 70 were selected using probability proportionate to size (PPS). The 70 Ilakas were proportionately distributed across Nepal’s three ecological belts; mountains (5 Ilakas), hills (30 Ilakas) and the Terai (35 Ilakas), based on the population proportion according to the National Population and Housing Census 2011 of Nepal [9]. Mountains refers to the high mountain Himalayan region, hills refers to adjacent region in a lower altitudinal belt while the Terai refers to the lowest terrain in the country ranging from 70 to 700 metres above the sea level. Individual wards in VDCs or municipalities were considered as clusters and these clusters were taken as the secondary sampling unit (SSU). A ward is the lowest level in the administrative division and each VDC is composed of 9 wards, whereas number of wards in each municipality ranges from 9 to 35. Three clusters were selected from each of the sampled Ilakas using the PPS sampling method, leading to the selection of 210 wards. Twenty households were selected from each cluster using systematic random sampling. One participant of the eligible candidates (15–69 years) in each selected household was selected to take part in the survey using the Kish method [14]. Of the 4,200 adults (15–69 years) targeted, we had 4143 adults participate in STEP I (response rate 98.6%), 4,124 in STEP II (response rate 98.2%) and 3,772 in STEP III (response rate 89.8%).

### Data Collection

The survey was conducted using the WHO NCD STEPS instrument version 2.2 [15], which prescribes three steps for measuring NCD risk factors. STEP I measures behavioural risk factors, STEP II covers physical measurements, and STEP III measures biological risk factors. Socio-demographic information on age and gender, education, marital and work status, as well as information on tobacco use, alcohol consumption, fruit and vegetable consumption, physical activity, and history of chronic conditions was collected by trained interviewers in face-to-face interviews. We used wet chemistry to measure biological risk factors.

#### Current smoking

Questions and pictorial show cards of tobacco products were used to identify current users (those who had smoked in the past 30 days).

#### Alcohol consumption

Questions were asked to determine the percentage of lifetime abstainers, past 12 months abstainers and current users of alcohol using the WHO protocol. Consumption of ≥60 gm of pure alcohol for men and ≥40 gm of pure alcohol for women on an average day in the past 30 days was considered harmful use [14]. To encourage respondents to disclose the alcohol and tobacco consumption habits, we maintained privacy during interviews and ensured respondents that responses would be reported anonymously.

#### Diet

Information was recorded on the number of days that respondents consumed fruit and vegetables in a typical week, and the number of servings of fruit and vegetables consumed on average per day. Less than five servings of fruits and vegetables per day was considered insufficient fruit and vegetable intake [14].

#### Physical activity

Physical activity was assessed using the Global Physical Activity Questionnaire (GPAQ) [16]. The GPAQ asks respondents about activity for transport purposes, vigorous and moderate activity at work, and vigorous and moderate activity in leisure time, and time spent sitting. Show-cards with culturally relevant examples were used to aid respondents in classifying activities. Analysis and categorization followed existing guidelines [15,17], and those who did not meet the criteria for vigorous and moderate intensity activities were categorised as having low physical activity.

#### History of raised blood pressure and blood glucose

Participants were asked about their history of raised blood pressure or blood glucose and treatment advised by a doctor to control these (such as medicines prescribed, a special diet to be followed, advice to reduce salt intake, lose weight, stop smoking, or do more exercise).

#### Physical measurements

Using the STEPS protocol and recommended instruments ensured accuracy of height and weight measurements, BMI calculations and blood pressure measurements. Height and weight were measured and body mass index (BMI) calculated according to the protocol. Height was recorded in centimetres using a portable standard stature scale. Weight was recorded in kilograms using a portable digital weighing scale (Seca, Germany). Waist and hip circumference was measured in centimetres using constant tension tapes (Seca, Germany) [14]. A BMI of ≥30.0 and between 25.0 and 29.9 was considered obese and overweight, respectively.

#### Blood pressure measurement

Blood pressure was measured using a digital, automated blood pressure monitor (OMRON digital device, OMRON, Netherlands) with an appropriate sized cuff. Raised blood pressure was defined as having systolic blood pressure ≥140 mm Hg and/or diastolic blood pressure ≥90 mm Hg during the study, or being previously diagnosed as having hypertension. This was determined by documentation such as a treatment record book, or participant history of medication for high blood pressure [14].

#### Biochemical measurements

A mobile laboratory was used in data collection. The mobile laboratory contained logistics and human resources required including a semi-auto analyser and all of the chemicals required for blood glucose testing and lipid profile measurements. For preservation of the chemicals used for the tests and ensuring that the cold chain was maintained for collected samples, continuous refrigeration was ensured using an electric generator. Fasting samples were taken to measure blood glucose and blood lipids and measured using the wet (liquid) method. Participants were instructed to fast overnight for 12 hours and diabetic patients on medication were reminded to bring their medicine/insulin with them and take their medicine after providing the blood sample. A venous blood sample (4 ml of blood) was taken using a flashback needle with an aseptic technique and kept in plain and fluoride treated tubes. Those samples were kept in an ice pack carrier and brought to the mobile laboratory within one hour. Biochemical measurements of blood glucose and lipids were done using semi-automated procedures (Bioanalyzer, Analyticon, Germany) and commercially available kits (Analyticon, Germany). Plasma glucose was estimated using the GOD-PAP (glucose oxidase/peroxidise–phenol-4-amenophenazone) method. Serum total cholesterol was determined by an enzymatic endpoint method using the CHOD-PAP (cholesteroloxidase/peroxidase– 4-phenol-aminoantipyrine) method [18]. Participants with blood glucose level ≥126 mg/dl or currently under medication for raised blood glucose were considered as having raised blood glucose. The lipid profile included total cholesterol, triglycerides and HDL cholesterol. LDL cholesterol calculated using the aforementioned three parameters of cholesterol. The cut off point for raised total cholesterol was ≥190 mg/dl. A team of medical laboratory technologists, medical laboratory technicians and pathologist used a predefined protocol including routine calibration for ensuring test accuracy.

Data was collected electronically using personal digital assistants (PDAs) programmed with WHO e-STEPS software.

### Data processing and analysis

Data collected on PDAs was downloaded onto computers using a Windows Mobile Device Centre. Files on each participant (questionnaire, body measurements, biochemical measurements and Kish data) were then merged using the participant identity (PID) number cross-checked with participant name and identification number. After merging, common variables in the dataset were matched and inconsistencies were corrected. Data cleaning was done using SPSS and analysis was done with STATA 12.0 SE version.

All estimates were weighted by sample weights and presented with 95% Confidence Intervals (CIs). Further stratification by individual characteristics (age, gender, education and marital status) and cluster characteristics (ecological zone and place of residence) were included in this analysis. Prevalence estimates with their 95% CIs were calculated using Taylor series linearization. Chi-square statistics were used to test associations between covariates and risk factors. Adjusted prevalence ratio (APR) was calculated using multiple Poisson regression, with all covariates (age, gender, education, marital status, ecological zone and place of residence) included simultaneously in the model in order to assess the determinants of NCD risk factors. The APRs rather than odds ratios allowed us to compare the relative strengths of association in a manner that was not biased by whether a risk factors was rare or common [19]. To reflect clustering within individuals, we considered the number of risk factors that each respondent had at the time of the survey (from 0 to 8) and examined the mean number and CIs of risk factors by covariates. We examined the independent effects of covariates on risk factor clustering within individuals by modeling a multiple Poisson regression, with the number of risk factors as the dependent variable. All the analysis carried out was using complex survey design; wards were considered as cluster and ecological zones as strata. A p-value <0.05 was considered as statistically significant.

### Ethical Considerations

This study was approved by the Ethical Review Board (ERB) of the Nepal Health Research Council (NHRC). Formal permission was taken from authorities in the selected districts, VDCs and municipalities. Informed written consent was obtained from all participants. In the case of minors (<18 years old), written consent was first obtained from the next of kin and then from all child participants. The objectives of the research were explained in simple language, and participants were also provided with an information sheet containing the research objectives, data collection methods, the role of participants, personal and community benefits, as well as any potential harm to participants. A participant feedback form was also provided to all participants after taking their physical and biochemical measurements. The confidentiality of the information gathered was maintained. Any waste generated during the laboratory procedures was properly disinfected using aseptic techniques and safely disposed of. All blood samples were discarded after completing biochemical measurements.

## Results

### Characteristics of participants enrolled in the study

The socio-demographic characteristics of the sample population are presented in "Table 1". The sample population was just over two thirds women (68% women; 32% men). The age range of the sample was 24% in the 15–29 years age group, 38% in the 30–44 years age group and 39% in the 45–69 years age group. The rural/urban makeup of participants was 82% from rural areas and 18% from urban areas. The proportion of respondents from the mountains, hills and Terai belts was similar to their national proportions, with about 50% from the Terai, 43% from the hill and 7% from the mountain region. Nearly half of participants (45%) did not have any formal schooling, and 86% were currently married.

**Table 1:** Characteristics of participants enrolled in the study.

Characteristics	Un-weighted number	Un-weighted Percent (%)	Weighted Percent (%)
**Age group**			
15–29	972	23.5	46.5
30–44	1,558	37.6	26.8
45–69	1,613	38.9	26.7
**Gender**			
Men	1,336	32.2	49.1
Women	2,807	67.8	50.9
**Level of education**			
No formal	1,851	44.7	30.6
Primary	1,021	24.6	25.4
Secondary	773	18.7	25.3
Higher	498	12.0	18.6
**Marital Status** *			
Never married	336	8.1	18.7
Currently married	3,567	86.1	78.0
Divorced/Widowed/Separated	237	5.8	3.3
**Ecological zone**			
Mountain	297	7.1	6.5
Hill	1,768	42.9	42.8
Terai	2078	50.0	50.6
**Place of residence**			
Rural	3,366	81.5	80.9
Urban	777	18.5	19.1

*2 refused to answer hence total response is 4,*141*

### Behavioural risk factors

#### Tobacco consumption

The overall prevalence of current smoking was 19% (95% CI: 16.6–20.6) "Table 2", with the highest prevalence among adults aged 45–69 years old (29%, 95% CI: 25.8–31.6). More men (27%) smoked than women (10%). The prevalence of smoking was higher in residents of rural areas (20%, 95% CI: 17.8–22.2) compared to those in urban areas (12%, 95% CI: 9.1–16.7), and higher among those who had primary education (26%, 95% CI: 21.7–30.2) compared to those who had higher education (10%, 95% CI: 6.5–13.8).

**Table 2:** Prevalence (%) of behavioral and biological risk factors for selected non-communicable diseases among aged 15–69 years.

	Un-weighted number (N)	Current smoking (95%CI)	Harmful use of alcohol (95%CI)	Insufficient fruit and vegetable intake (95%CI)	Low physical activity (95%CI)	Overweight or obesity (95%CI)	Raised blood pressure (95%CI)	Raised blood glucose (95%CI)	Raised total cholesterol (95%CI)
**Age group**									
**15–29**	**972**	11.4 (8.9–14.6)	1.1 (0.4–2.8)	99.0 (97.8–99.5)	2.3 (1.3–4.0)	13.4 (11.1–16.0)	13.3 (10.7–16.4)	0.9 (0.4–2.0)	14.3 (11.4–17.7)
**30–44**	**1,558**	20.7 (17.6–24.2)	2.9 (1.7–5.0)	99.0 (97.8–99.5)	2.5 (1.5–4.0)	29.8 (26.2–33.7)	26.6 (23.3–30.1)	3.1 (2.0–4.6)	26.3 (23.0–29.9)
**45–69**	**1,613**	28.6 (25.8–31.6)	2.7 (1.8–4.1)	98.7 (97.7–99.2)	6.4 (5.0–8.0)	26.9 (23.7–30.4)	46.7 (43.4–50.0)	8.7 (7.1–10.6)	33.0 (29.7–36.4)
***P*-value**		<0.001	0.082	0.787	<0.001	<0.001	<0.001	<0.001	<0.001
**Gender**									
**Men**	**1,336**	27.0 (23.8–30.5)	3.1 (2.1–4.6)	98.9 (97.8–99.4)	4.5 (3.3–6.1)	21.0 (18.2–24.3)	31.1 (27.8–34.6)	4.6 (3.5–5.9)	24.4 (21.4–27.7)
**Women**	**2,807**	10.3 (8.8–12.0)	0.9 (0.5–1.6)	98.9 (98.1–99.4)	2.4 (1.9–3.1)	21.8 (19.5–24.2)	20.6 (18.6–22.8)	2.7 (2.0–3.6)	20.9 (18.7–23.3)
***P*-value**		<0.001	<0.001	0.889	0.001	0.661	<0.001	0.004	0.040
**Level of education**									
**No formal schooling**	**1,851**	22.1 (19.3–25.1)	1.9 (1.2–3.0)	99.6 (98.6–99.9)	3.9 (2.9–5.1)	18.8 (16.1–21.8)	29.6 (26.5–32.8)	3.1 (2.3–4.2)	22.5 (19.8–25.4)
**Primary**	**1,021**	25.7 (21.7–30.2)	2.9 (1.7–4.9)	99.1 (98.1–99.6)	3.2 (2.1–4.9)	21.8 (18.5–25.5)	24.8 (21.4–28.6)	4.5 (3.1–6.4)	22.2 (18.8–25.9)
**Secondary**	**773**	13.5 (10.4–17.3)	2.2 (1.0–4.7)	99.0 (97.4–99.6)	2.6 (1.4–4.9)	23.2 (19.5–27.4)	23.5 (19.5–28.0)	3.1 (2.0–4.6)	22.2 (18.4–26.6)
**Higher**	**498**	9.5 (6.5–13.8)	0.7 (0.1–4.4)	97.4 (97.4–98.7)	4.0 (2.4–6.6)	22.8 (18.6–27.6)	23.7 (19.1–29.1)	4.0 (2.6–6.1)	24.0 (19.1–29.7)
***P*-value**		<0.001	0.287	0.012	0.562	0.212	0.073	0.292	0.900
**Marital status**									
**Never married**	**336**	10.2 (6.8–14.9)	0.7 (0.1–4.7)	98.3 (95.6–99.4)	3.0 (1.4–6.2)	8.2 (5.4–12.1)	13.4 (9.6–18.5)	1.4 (0.5–4.2)	12.7 (8.4–18.6)
**Currently married**	**3,568**	20.0 (17.9–22.3)	2.3 (1.6–3.3)	99.0 (98.4–99.4)	3.3 (2.6–4.3)	24.5 (22.1–27.2)	28.0 (25.7–30.5)	4.0 (3.2–5.0)	24.8 (22.5–27.3)
**Divorced/Widowed/Separated**	**237**	29.1 (21.7–37.8)	2.4 (0.9–6.8)	98.7 (95.8–99.6)	8.1 (5.0–12.7)	22.9 (17.1–30.0)	41.1 (33.6–49.1)	6.9 (3.1–14.5)	26.0 (19.8–33.3)
***P*-value**		<0.001	0.224	0.404	0.099	<0.001	<0.001	0.042	<0.001
**Ecological zone**									
**Hill**	**1,800**	19.9 (16.9–23.2)	3.1 (2.0–5.0)	98.7 (97.6–99.3)	2.2 (1.4–3.5)	26.2 (22.5–30.3)	25.9 (22.4–29.6)	3.5 (2.4–5.2)	21.7 (18.6–25.1)
**Mountain**	**300**	24.6 (15.0–37.5)	5.7 (2.1–14.7)	99.3 (96.9–99.8)	0.5 (0.1–1.9)	9.0 (4.6–17.0)	22.2 (16.8–28.8)	1.9 (0.5–6.4)	14.1 (10.0–19.3)
**Terai**	**2,100**	16.5 (14.2–19.1)	0.6 (0.3–1.0)	99.0 (98.0–99.5)	4.8 (3.7–6.3)	18.9 (16.4–21.7)	26.1 (23.2–29.2)	3.9 (3.1–4.9)	24.5 (21.3–27.9)
***P*-value**		0.147	<0.001	0.760	<0.001	<0.001	0.630	0.495	0.028
**Place of residence**									
**Rural**	**3,422**	19.9 (17.8–22.2)	2.3 (1.5–3.4)	99.3 (98.7–99.6)	3.1 (2.4–4.1)	19.2 (17.0–21.6)	24.9 (22.7–27.3)	2.9 (2.3–3.8)	22.0 (19.6–24.5)
**Urban**	**778**	12.4 (9.1–16.7)	0.9 (0.4–2.3)	97.1 (94.8–98.4)	4.8 (3.1–7.4)	30.9 (25.4–37.1)	29.1 (23.7–35.2)	6.5 (4.5–9.1)	25.3 (20.6–30.7)
***P*-value**		0.003	0.065	<0.001	0.101	<0.001	0.173	<0.001	0.234
**Total**	**4,143**	18.5 (16.6–20.6)	2.0 (1.4–2.9)	98.9 (98.3–99.3)	3.4 (2.7–4.3)	21.4 (19.3–23.7)	25.7 (23.6–28.0)	3.6 (2.9–4.5)	22.6 (20.5–24.9)

*Note*: *P*-values are for test for differences in prevalence

#### Alcohol consumption

Harmful alcohol consumption was observed in 2% (95% CI, 1.4–2.9) of participants "Table 2". A higher prevalence was observed among men (3%, 95% CI: 2.1–4.6) compared to women (1%, 95% CI: 0.5–1.6), and in mountains (6%, 95% CI: 2.1–14.7) compared to the Terai (<1%, 95% CI: 0.3–1.0).

#### Fruits and vegetables intake

Almost the entire study population (99%) had insufficient fruit and vegetable intake according to WHO recommendations "Table 2". Urban respondents had a slightly lower prevalence of insufficient fruit and vegetable intake (97%, 95% CI: 94.8–98.4) compared to those from rural areas (99%, 95% CI: 98.7–99.6).

#### Physical activity

Low physical activity was prevalent among 3% (95% CI: 2.7–4.3) of the respondents "Table 2". Those aged 45–69 had the highest prevalence (6%, 95% CI: 5.0–8.0). Compared to women (2%, 95% CI: 1.9–3.1), men had a higher prevalence of low physical activity (5%, 95% CI: 3.3–6.1). Likewise, low physical activity was more prevalent in Terai residents (5%, 95% CI: 3.7–6.3) compared to mountain residents (<1%, 95% CI: 0.1–1.9).

### Biological risk factors

#### Body mass index

Overweight and obesity combined was observed in 21% of participants "Table 2". Almost 30% (95% CI: 26.2–33.7) of respondents aged 30–44 years had a body mass index ≥ 25 kg/m^2^. A higher prevalence of overweight and obesity was observed among respondents who resided in the hills (26%, 95% CI: 22.5–30.3), urban areas (31%, 95% CI: 25.4–37.1) and those currently married (25%, 95% CI: 22.1–27.2) compared to those who resided in the mountains (9%, 95% CI: 4.6–17.0), rural areas (19%, 95% CI: 17.0–21.6) and those never married (8%, 95% CI: 5.4–12.1), respectively.

#### Blood pressure

The prevalence of raised blood pressure, including those who were on medication for hypertension was 26% (Table 2). Higher prevalence was observed among those aged 45–69 years (47%, 95% CI: 43.4–50.0), men (31%, 95% CI: 27.8–34.6), and those divorced/widowed/separated (41%, 95% CI: 33.6–49.1) compared to those aged 15–29 years (13%, 95% CI: 10.7–16.4), women (21%, 95% CI: 18.6–22.8) and those never married (13%, 95% CI: 9.6–18.5), respectively.

#### Blood glucose

Four percent of the study participants had raised blood glucose "Table 2". The prevalence was higher among those aged 45–69 years (9%, 95% CI, 7.1–10.6) and residents from urban areas (7%, 95% CI: 4.5–9.1) compared to those aged 15–29 years (1%, 95% CI: 0.4–2.0) and those from rural areas (3%, 95% CI: 2.3–3.8) respectively.

#### Serum cholesterol

Raised total cholesterol was observed in 23% (95% CI: 20.5–24.9)of participants "Table 2". Higher prevalence was observed among those aged 45–69 years (33%, 95% CI: 29.7–36.4), inhabitants of the Terai (25%, 95% CI: 21.3–27.9), and among those divorced/widowed/separated (26%, 95% CI: 19.8–33.3) compared to those aged 15–29 years (14%, 95% CI: 11.4–17.7) inhabitants of mountains (14%, 95% CI: 10.0–19.3) and among those never married (13%, 95% CI: 8.4–18.6), respectively.

### Determinants of behavioral and biological risk factors

The APR for determinants of behavioral and biological risk factors is presented in "Table 3". The study revealed a lower prevalence of smoking among women than men (APR: 0.3; 95% CI: 0.25–0.36), and in those who had higher education levels compared to those with no formal education (APR: 0.39; 95% CI: 0.26–0.58). Harmful alcohol use was lower in women than men (APR: 0.26; 95% CI: 0.14–0.48), and in Terai residents compared to hill residents (APR: 0.16; 95% CI: 0.07–0.36).

**Table 3:** Determinants of behavioral and biological risk factors for selected non-communicable diseases among aged 15–69 years.

	Current smoking APR(95%CI)	Harmful use of alcohol APR(95%CI)	Insufficient fruit and vegetable intake APR(95%CI)	Physical inactivity APR(95%CI)	Overweight or obesity APR(95%CI)	Raised blood pressure APR(95%CI)	Raised blood glucose APR(95%CI)	Raised total cholesterol APR(95%CI)
**Age group**								
**15–29**	1	1	1	1	1	1	1	1
**30–44**	1.36 (1.01–1.85)*	1.81 (0.65–5.04)	0.99 (0.98–1.01)	1.28 (0.57–2.87)	2.01 (1.60–2.52)*	1.95 (1.49–2.56)*	4.63 (1.82–11.76)**	1.86 (1.43–2.41)*
**45–69**	1.62 (1.19–2.20)*	1.60 (0.62–4.10)	0.99 (0.98–1.00)	3.08 (1.43–6.64)*	2.02 (1.60–2.55)*	3.47 (2.65–4.55)*	15.62 (6.31–38.63)*	2.54 (1.96–3.29)*
**Gender**								
**Men**	1	1	1	1	1	1	1	1
**Women**	0.30 (0.25–0.36)*	0.26 (0.14–0.48)*	0.99 (0.98–1.01)	0.55 (0.38–0.80)*	1.19 (1.02–1.39)*	0.69 (0.60–0.80)*	0.86 (0.58–1.28)	0.97 (0.83–1.14)
**Ecological zone**								
**Hill**	1	1	1	1	1	1	1	1
**Mountain**	1.01 (0.70–1.44)	1.44 (0.49–4.21)	0.99 (0.98–1.01)	0.28 (0.07–1.12)	0.41 (0.21–0.80)*	0.91 (0.67–1.23)	0.84 (0.25–2.84)	0.72 (0.49–1.05)
**Terai**	0.79 (0.64–0.99)*	0.16 (0.07–0.36)*	1.00 (0.99–1.01)	2.43 (1.46–4.07)*	0.80 (0.67–0.96)*	1.08 (0.91–1.28)	1.50 (0.97–2.30)	1.23 (0.99–1.51)
**Place of residence **								
**Rural**	1	1	1	1	1	1	1	1
**Urban**	0.73 (0.52–1.01)	0.46 (0.14–1.45)	0.98 (0.96–1.00)	1.57 (0.89–2.75)	1.39 (1.15–1.67)*	1.14 (0.92–1.41)	2.05 (2.29–3.25)*	1.03 (0.81–1.31)
**Level of education **								
**No formal schooling**	1	1	1	1	1	1	1	1
**Primary**	0.93 (0.78–1.11)	1.09 (0.63–1.89)	0.99 (0.98–1.00)	0.92 (0.57–1.49)	1.46 (1.21–1.76)*	1.00 (0.86–1.18)	2.30 (1.48–3.59)*	1.25 (1.04–1.50)*
**Secondary**	0.54 (0.41–0.70)*	1.06 (0.49–2.31)	0.98 (0.97–1.01)	0.79 (0.43–1.44)	1.85 (1.49–2.29)**	1.14 (0.93–1.39)	2.11 (1.18–3.79)*	1.47 (1.18–1.82)*
**Higher education**	0.39 (0.26–0.58)*	0.38 (0.07–2.03)	0.97 (0.95–0.99)	1.30 (0.70–2.40)	1.94 (1.51–2.47)*	1.24 (1.00–1.55)	3.11 (1.65–5.87)*	1.76 (1.35–2.28)*
**Marital status **								
**Never married**	1	1	1	1	1	1	1	1
**Currently married**	1.32 (0.81–2.16)	2.21 (0.41–12.03)	1.00 (0.99–1.02)	0.83 (0.32–2.13)	2.31 (1.47–3.63)*	1.24 (0.84–1.83)	0.78 (0.24–2.59)	1.43 (0.94–2.16)
**Divorce/Widow/Separated**	1.51 (0.85–2.67)	1.93 (0.23–16.48)	1.00 (0.98–1.03)	1.61 (0.57–4.54)	2.22 (1.35–3.65)*	1.36 (0.87–2.12)	1.03 (0.23–4.64)	1.32 (0.80–2.16)

*statistically significant at p<0.05

Physical inactivity was significantly lower among women than men (APR: 0.55; 95% CI: 0.38–0.80), however women had a significantly higher prevalence of being overweight or obese compared to men (APR: 1.19; 95% CI: 1.02–1.39). Being overweight or obese was significantly less prevalent in mountain residents than in hill residents (APR: 0.41; 95% CI: 0.21–0.80). Urban residents were more overweight or obese in comparison to rural residents (APR: 1.39; 95% CI: 1.15–1.67).

A lower prevalence of raised blood pressure was observed among women than men (APR: 0.69; 95% CI: 0.60–0.80). A higher prevalence of raised blood glucose was observed among urban residents compared to rural residents (APR: 2.05; 95% CI: 1.29–3.25). Total cholesterol level was significantly higher among 45–69 year olds in comparison to those aged 15–29 years (APR: 2.54; 95% CI: 1.96–3.29). Similarly, a higher prevalence of raised total cholesterol level was observed among those respondents having higher education levels compared to those respondents who had no formal education (APR: 1.76; 95% CI: 1.35–2.28).

### Mean number of behavioral or biological risk factors

**Fig 1:**
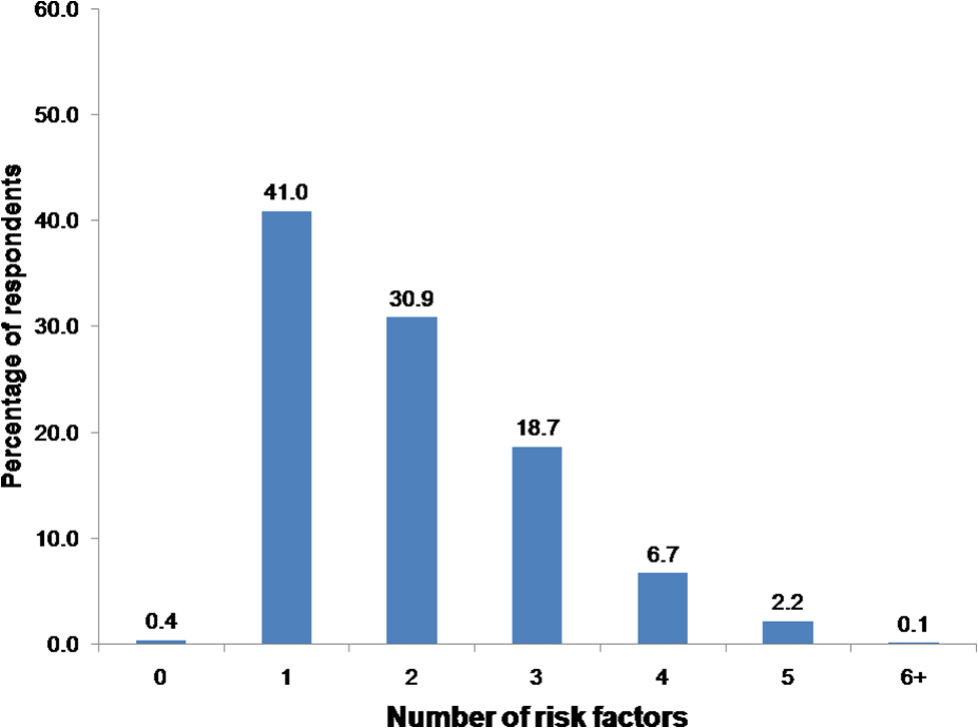
Prevalence of multiple risk factors for non-communicable diseases among aged 15–69 years.

Only 0.4% of the population was completely free from the eight established risk factors "Fig 1". Nearly half of respondents had at least one risk factor, one third had 2 risk factors, nearly one fifth had 3 risk factors and almost one tenth of respondents had 4 or more risk factors. This study found on average that respondents demonstrated 2 NCD risk factors (95% CI: 1.92–2.03) "Table 4".

**Table 4:** Mean number of behavioral or biological risk factors for non-communicable diseases and independent effects of covariates on risk factor clustering in individuals.

	Mean number of risk factors (95%CI)	ARR^a^ (95%CI)
**Age group**		
15–29	1.56 (1.48–1.63)	1
30–44	2.12 (2.04–2.21)	1.32 (1.23–1.42)*
45–69	2.53 (2.45–2.61)	1.58 (1.47–1.70)*
**Gender**		
Men	2.16 (2.08–2.25)	1
Women	1.80 (1.75–1.85)	0.84 (0.80–0.88)*
**Ecological zone**		
Hill	2.04 (1.94–2.14)	1
Mountain	1.78 (1.61–1.94)	0.88 (0.80–0.97)*
Terai	1.94 (1.87–2.01)	0.98 (0.93–1.03)
**Place of residence**		
Rural	1.95 (1.89–2.01)	1
Urban	2.06 (1.91–2.21)	1.04 (0.97–1.12)
**Level of education**		
No formal schooling	2.04 (1.96–2.11)	1
Primary	2.04 (1.94–2.15)	1.08 (1.03–1.14)*
Secondary	1.92 (1.81–2.02)	1.09 (1.03–1.16)*
Higher education	1.86 (1.74–1.98)	1.10 (0.97–1.19)
**Marital status**		
Never married	1.49 (1.39–1.59)	1
Currently married	2.07 (2.01–2.13)	1.16 (1.08–1.23)*
Divorce/Widow/Separated	2.42 (2.21–2.62)	1.23 (1.08–1.39)*
Total	1.97 (1.92–2.03)	

*statistically significant confidence interval

^a^ The number of risk factors was the dependent variable. Each RR reflects the risk of having *x* or more risk factors versus having fewer against the risk in the reference group. Hence, the ARR represents the average effect of the covariate on the risk of having *x* number of risk factors or more.

“Table 4” presents the independent effect of various covariates on the clustering of risk factors at the individual level, as revealed by multiple Poisson regression. The age, gender, level of education, marital status and ecological belts were significantly associated with mean number of risk factors. Women (Adjusted Relative Risk (ARR): 0.84; 95%CI: 0.80–0.88) and inhabitants of mountains (ARR: 0.88; 95%: 0.80–0.97) had significantly fewer risk factors than men and inhabitants of hills respectively.

## Discussion

This national survey assessed the prevalence and determinants of major NCD risk factors in Nepal–both modifiable behavioural risk factors (current smoking, alcohol consumption, low fruit and vegetable consumption, and physical inactivity) and biological risk factors (overweight, obesity, raised blood pressure, raised blood glucose and abnormal lipids). This study has demonstrated that Nepal has a particularly high prevalence of certain NCD risk factors, such as current smoking, low fruit and vegetable consumption, raised blood pressure and abnormal lipids. In addition, there is wide variation in prevalence by age group, gender, place of residence and ecological zone.

### Behavioural risk factors

The prevalence of current smoking (19%) is consistent with the 2007/08 NCD risk factors survey 24% (95% CI: 16–31). The prevalence is high in comparison to neighbours India (14%) and Sri Lanka (15%) [20]. However, it is lower than that in China (28%), Bangladesh (23%), Vietnam (24%), Thailand (24%) and Russia (39%) [20].This highlights the importance of strong implementation and monitoring of the comprehensive tobacco control law introduced in 2011. This law includes provision of at least 75% tobacco packaging with pictorial health warning, a ban on smoking in public places, work places, and public transport, a ban on all types of tobacco advertising, promotion and sponsorship, and provision of health fund for tobacco control. Due to tobacco industry litigation, implementation of the pictorial health warning was implemented only in 2014.

In addition to smoking, 18% of respondents (31% of men and 5% of women) consumed smokeless tobacco [21] with 31% of the population (men 48% and women 14%) using either smoke or smokeless forms of tobacco [21]. Tobacco consumption is high among men and low among women, which could be due to the social unacceptability of women’s use of tobacco. Tobacco use in Nepal is much less than current tobacco use in Bangladesh and Myanmar, where around 32% and 30% of the adult population, respectively, consume smokeless tobacco, and half of the adult population consume either form of tobacco [22,23]. On the backdrop of global targets to bring tobacco consumption down to less than 5% by 2040, from the UN high level meeting on NCDs in 2011 [24], tobacco consumption in Nepal appears to be substantially high, posing a challenge ahead. Another cause for concern is that more than one third of study respondents were exposed to second hand smoke at home (36.1%) or in the workplace (37.2%). This presents the need of improving people's behaviour as it appears to be not changing even when the law exists on ban of use of tobacco in public places including work places and at home.

Harmful use of alcohol along, low physical activity levels and raised blood glucose levels have been observed as the least frequent NCD risk factors in Nepal. Harmful use of alcohol, though prevalent in only about 2% of the total study population, is more than twice as high among those aged 30 years and above, compared to those aged 15–29. The proportion of the total population with harmful use of alcohol was not available in previous national survey. However, the proportion of men with harmful use of alcohol among the current drinkers has dropped from 32.3% in 2007/08 to 11.1% in 2012/13 [8]. The observed difference however could be due to the difference in sampling design between the surveys. The 2007/08 NCD risk factors survey collected information from 15 districts of Nepal and oversampled urban clusters without adjusting sample weights (50% urban clusters despite having a 17% urban population nationally). The higher prevalence reported in the previous survey may thus be due to a higher representation of urban clusters. In contrast, the proportion of women with harmful use among the current drinkers has increased from 9.9% in 2007/08 to 13.2% in 2012/13. However, this change was not statistically significant [8]. The factors affecting the reduction in harmful use of alcohol in men warrants further examination, by analysing the determinants of harmful alcohol use in both studies.

The current study also revealed that 17% of respondents were current drinkers, which indicates a likelihood to turn into category III drinkers (harmful use). In comparison to geographical neighbours, this is much lower than the prevalence of current drinkers in Kerala, India where 41% of the population were found to be current drinkers in a study published in 2008 [25]. In contrast, only 0.8% of the population in Bangladesh were found to be current drinkers by the STEPS survey carried out in 2009/10, [22] while there were about 12.9% of current drinkers in Myanmar as per the 2009 STEPS survey [23]. People most often consume local home brewed alcoholic beverages in Nepal which is considered part of their tradition and hence appears to be a big challenge ahead to reduce its consumption and thus preventing the population from its adverse effects [8,26].

The current study findings on low prevalence of low physical activity prevalent is in line with the findings of the world health survey, as physical inactivity was found to be less prevalent in populations of low socio-economic status, especially in low-income countries [27].

Another serious concern in Nepal is the extremely high (99%) prevalence of insufficient intake of fruit and vegetables. This suggests an urgent need for public health interventions, although local context needs to be particularly considered for this risk factor. This finding is similar to the findings of another study conducted among in a peri-urban population in Nepal, which found that about 98% of the study population consumed less than the recommended 5 servings daily of fruits and vegetables [28]. The prevalence of low fruit and vegetable intake is increasing compared to the previous STEPS survey in 2007/08, however, again this difference could be due to a difference in sampling methods between the two surveys [8] as there are contradictory reports of increased per capita vegetable consumption in the last two decades [29].

### Biological risk factors

A high prevalence of being overweight and obesity (21%) has been observed in this study, which is even higher (31%) among urban residents. Although physical inactivity prevalence is low, insufficient fruit and vegetables intake could be a contributing factor. This indicates a need for further analysis of the current data, beyond the scope of the current analysis. The combined prevalence of being overweight and obese has increased dramatically from a modest figure of 8.9% in 2007/08 [8]. The higher prevalence of being overweight among urban residents is in consistent with results from a systematic review of trends and socio-economic factors of obesity in South Asia [30]. The prevalence among urban residents found in the current study is similar to that of a study in Gujarat, India [31] but higher than of a study among the urban population in Karachi, Pakistan [32]. However it is still low compared to high-income countries such as Australia, England and the United States [33,34,35].

One in every four study respondents had raised blood pressure in Nepal. Recent evidence of the rising prevalence of hypertension in the Nepalese community from another study underscores that this is a key risk factor [36]. In 2010, high blood pressure was one of the three leading risk factors for global disease burden and was the leading risk factor for most countries in Asia, North Africa and the Middle East [37]. In comparison with the previous national survey in 2007/08, the prevalence of raised blood pressure has decreased from 31.3% [8] to 25.7% in 2012/13. The prevalence of raised blood pressure in Nepal found by the current study is slightly higher than that found in the Maldives (17%) in a similarly aged population [38]. The prevalence of raised blood pressure is even higher among some sub-populations in Nepal, such as those aged 45 to 69 years (47%), men (31%) and urban residents (29%). Furthermore, almost 90% of respondents with raised blood pressure were not currently under any treatment for raised blood pressure, which indicates a high unmet need for prevention and management actions. A lack of policy and insufficient programs on NCDs and its risk factors could be a key contributing factor to this situation. In the current study, tobacco smoking, alcohol drinking and raised blood pressure (BP) were more frequent in males than females which is consistent with findings from a STEPS Survey conducted in Malawi [39].

In line with previous studies, the observed prevalence of raised blood glucose levels in the current study was 4%, which is less than estimates for South Asia overall (8%) [40]. However, a higher prevalence has been observed among respondents aged 45 to 69 years in Nepal. This suggests that health services, including primary care services, should be equipped down to the primary care level for the management of diabetes cases including services for screening, diagnosis, treatment and follow up. In the global context, the prevalence observed in the current study is less than that seen in rural India (6% among men and 5% among women) in a study carried out among 1600 rural villages and published in 2010 [41], and is higher than that seen in Vietnam (1% among men and 1.1% among women) [42]. A population based study from a rural Ugandan district in 2012 showed an overall prevalence of 2.9% and in Malawi a diabetes prevalence of 5.6% was seen [43,44]. The combined prevalence of indicators; raised blood glucose (≥126 mg/dl) and impaired fasting glycaemia (≥110 mg/dl and x126 mg/dl) is as high as 7.7% with nearly one tenth for men in the current study. A study analysing the data from the national surveillance of NCD risk factors survey of Iran from 2005 to 2007 showed that impaired fasting glycaemia and even lower levels of fasting plasma glucose (90 mg/dl) are associated with a high prevalence of cardiovascular risk factors in Iranian adults and also suggests the FPG cut-off to be revised at 90 mg/dl to identify people with increased cardiovascular risk [45]. This suggests a potentially higher risk of cardiovascular disease among Nepalese adults as well.

### Demographic variation in risk factors

Most risk factors, such as current smoking, harmful alcohol consumption, raised blood pressure, and low physical activity, were more prevalent among men than women. However, obesity was more prevalent among women, even though more women than men achieved recommended physical activity levels. Such trends have been seen in previous studies in Nepal [46]. A previous study among the urban poor in Kathmandu, Nepal found a high prevalence of behavioural risk factors [47]. An Indian study showed that women have a poorer dietary pattern than men, a reflection of poor social status; this could also be true in Nepal as well given the socio-cultural and gender status similarities to its neighbour [48]. Compared to the hills, a lower prevalence of harmful alcohol use was observed in the Terai. A possible explanation is the low cultural acceptance of alcohol consumption in the Terai as compared to the hills and mountains.

Even though the prevalence of raised blood pressure was low (13%) among those aged 15–29 years, it was high among 30–44 year olds (27%) and even higher among those aged 45–69 years (47%), with similar patterns among men and women. Similar to other studies in the region, the current smoking, low physical activity, overweight or obesity, raised blood pressure, raised blood glucose and abnormal lipids levels were of a higher prevalence among older respondents (aged 45–69 years) than younger ones [31,37]. In a similar study from Gujarat, India, 29% of urban residents and 15% of rural residents were found to have raised blood pressure, respectively [31]. Both studies’ findings are consistent with the current study. A study in a rural Ugandan district, found the prevalence of raised blood pressure to be 22% for men, which is slightly lower than the current study and 21% for women which is similar to the findings of current study [43].

The prevalence of raised total cholesterol as found in the current study is also alarming, with more than one-fifth of the adult population having raised total cholesterol. This could be attributed to dietary patterns, genetic susceptibility and other factors not assessed in this study; this suggests the need for further investigation. As with raised blood pressure, the prevalence of raised total cholesterol was higher among the older age groups (30–44 years and 45–69 years).

### Number of behavioural or biological risk factors

Only <1% of the study population was found to be free of all studied NCD risk factors. This indicates that the burden of NCDs is likely to increase in the future if it is not addressed promptly by the stakeholders, including the Government of Nepal. Based on risk factors (current smoking, raised blood pressure, raised blood glucose and raised total cholesterol), the proportion of 40–69 year old adults with a 10-year risk of cardiovascular disease ≥30% was also substantial at 3.2%, with the proportion almost double (6.1%) among the 55–69 year old age group.

This STEPS survey suggests that NCD risk factors are quite prevalent among the Nepalese population, with variation by demographic factors. In addition, the proportion of deaths due to NCDs in Nepal is estimated to have risen from 51% in 2010 to 60% in 2014 [2,3]. There has been negligible action taken to prevent and control NCDs and their risk factors in Nepal so far. Preventing and controlling NCD risk factors is easier and less costly than treating NCDs, and a multi-sectoral approach to reduce the impact of NCDs on morbidity and mortality in Nepal is imperative over the next decade. As there are substantial subgroup differences in the APRs of risk factors such as current smoking, being overweight or obese and raised blood pressure, tailored, targeted interventions are necessary. For examples targeted interventions to: reduce smoking for rural populations and those with low levels of education; reduce harmful use of alcohol for residents of mountains; reduce raised blood glucose; reduce levels of being overweight or obese for urban populations; and interventions to reduce raised total cholesterol for residents of the Terai.

In conclusion, this study provides the first, and most comprehensive, national level evidence on the magnitude of NCD risk factors in the country of Nepal. The findings of this study are invaluable to support advocacy and formulation of NCD policy and plan of action in Nepal.

## Supporting Information

S1 Dataset Raw data of the NCD Risk Factors: STEPS Survey Nepal 2013. (XLSX)

S1 Codebook STEPS Instruments Nepal 2013 (Code Book). (PDF)

S2 Codebook Codebook: Extra variables not in STEPS Instruments. (XLSX)
