# Supplementary material for: The Burden and Determinants of Non Communicable Diseases Risk Factors in Nepal: Findings from a Nationwide STEPS Survey
Source: PLoS One. 2015 Aug 5;10(8):e0134834. doi: 10.1371/journal.pone.0134834 (PMC4526223; doi:10.1371/journal.pone.0134834)
Supplement: S1 Codebook — (PDF) [file pone.0134834.s002.pdf]

Annex III. Survey Instruments

WHO STEPS Instrument

for Non Communicable Diseases Risk Factor Surveillance

Nepal

Survey information

Licatuion and date		Response	Code
1	**Ward ID**	└─┴─┴─┘	I1
2	**Ward Number**	└─┴─┘	I2
3	**Interviewer ID**	└─┴─┴─┘	I3
4	**Date of completion of the instrument**	└─┴─┘ └─┴─┘ └─┴─┴─┴─┘<br>**dd** **mm** **year**	I4

Participant Id number └─┴─┘ └─┴─┘ └─┴─┴─┘ └─┴─┴─┘

Conset, interview language and name		Response	Code
5	Consent has been read and obtained	Yes 1<br>No 2 **If NO, END**	I5
6	Interview language	English 1<br>Nepali 2	I6
7	Time of interview<br>(24 hour clock)	└─┴─┘ : └─┴─┘<br>hrs mins	I7
8	Family surname		I8
9	First name		I9
**Additional information that may be helpful**			
10	Contact phone number where possible		I10

Record and file identification information (I5 to I10) separately from the completed questionnaire.

STEP I. Demographic information			
**Demographic information**			
**Question**		**Response**	**Code**
11	Sex (*record male female as observed)*	Male 1<br>Female 2	C1
12	What is your date of birth?<br>*Don't know 77 77 7777*	└─┴─┘ └─┴─┘ └─┴─┴─┴─┘ *If known, Go to C4*<br>dd mm year	C2
13	How old are you?	Years └─┴─┘	C3
14	In total, how many years have you spent in school or full-time study (excluding pre-school)?	Years └─┴─┘	C4
15	What is the **highest level of education** you have completed?	No formal schooling 1<br>Less than primary school 2<br>Primary school completed 3<br>Secondary school completed 4<br>Higher secondary (10+2)/ PCL completed 5<br>Bachelor degree completed 6<br>Post graduate degree 7<br>Refused 88	C5
16	What is your **ethnic background**?<br>(USE CASTE CLASSIFICATION CARD)	Dalit 1<br>Disadvantaged Janajatis 2<br>Disadvantaged non-Dalit Terai caste groups 3<br>Religious minorities 4<br>Relatively advantaged Janajatis 5<br>Upper caste groups 6<br>Refused 88	C6

Demographic information continued			
Question		Response	Code
17	What is your **marital status**?	Never married 1<br>Currently married 2<br>Separated 3<br>Divorced 4<br>Widowed 5<br>Cohabitating 6<br>Refused 88	C7
18	Which of the following best describes your **main work** status over the past 12 months?	Government employee 1<br>Non-government employee 2<br>Self-employed 3<br>Non-paid 4<br>Student 5<br>Homemaker 6<br>Retired 7<br>Unemployed (able to work) 8<br>Unemployed (unable to work) 9<br>Refused 88	C8
19	How many people older than 15 years, including yourself, live in your household?	Number of people └─┴─┘	C9

STEP I. Behavioural measurements

Tobacco use

Now I am going to ask you some questions about tobacco use.

	Question	Response	Code
20	Do you **currently** smoke any **tobacco** products, such as cigarettes, cigars, pipes, bidis, hukahs or tamakhus?<br><br>(USE SHOWCARD)	Yes 1<br>No 2 *If No, go to T8*	T1
21	Do you currently smoke tobacco products **daily**?	Yes 1<br>No 2	T2
22	How old were you when you **first started** smoking?	Age (years) __ __ *If known, go to T5a/T5aw*<br>Don't know 77	T3
23	Do you remember how long ago it was?(RECORD ONLY 1, NOT ALL 3)<br><br>*Don't know 77*	In Years __ __ *If known, go to T5a/T5aw*<br>OR in months __ __ *If known, go to T5a/T5aw*<br>OR in weeks __ __	T4a<br>T4b<br>T4c
24	On average, **how many** of the following products do you smoke **each day/week?**<br><br>(IF LESS THAN DAILY, RECORD WEEKLY)<br><br>(RECORD FOR EACH TYPE, USE SHOWCARD)<br><br>*Don't know 7777*	DAILY↓ WEEKLY↓<br>Manufactured cigarettes __ __ __ __ / __ __ __ __<br>Hand-rolled cigarettes __ __ __ __ / __ __ __ __<br>Pipes full of tobacco __ __ __ __ / __ __ __ __<br>Cigars, cheroots, cigarillos __ __ __ __ / __ __ __ __<br>Other __ __ __ __ / __ __ __ __<br>*If other, go to T5other, else go to T6*<br>Other (please specify): __ __ __ __ __ __	T5a/T5aw<br>T5b/T5bw<br>T5c/T5cw<br>T5d/T5dw<br>T5e/T5ew<br>T5other/<br>T5otherw
25	During the past 12 months, have you tried to **stop smoking**?	Yes 1<br>No 2	T6
26	During any visit to a doctor or other health worker in the past 12 months, were you advised to quit smoking tobacco?	Yes 1 *If T2=Yes, go to T12; if T2=No, go to T9*<br>No 2 *If T2=Yes, go to T12; if T2=No, go to T9*<br>No visit during the past 12 months 3 *If T2=Yes, go to T12; if T2=No, go to T9*	T7
27	In the past, did you **ever smoke** any tobacco products? (USE SHOWCARD)	Yes 1<br>No 2 *If No, go to T12*	T8
28	In the past, did you **ever** smoke **daily**?	Yes 1 *If T1=Yes, go to T12, else go to T10*<br>No 2 *If T1=Yes, go to T12, else go to T10*	T9

Tobacco use continued			
**Question**		**Response**	**Code**
29	How old were you when you **stopped** smoking?	Age (years) └─┴─┘ *If known, go to T12*<br>Don't know 77	T10
30	How **long ago** did you stop smoking?<br>(RECORD ONLY 1, NOT ALL 3)<br>*Don't know 77*	Years ago └─┴─┘ *If known, go to T12*	T11a
		OR Months ago └─┴─┘ *If known, go to T12*	T11b
		OR Weeks ago └─┴─┘	T11c
31	Do you **currently use** any **smoke-less tobacco** products such as *[snuff, chewing tobacco, nasal snuff, khaini, surti, gutka]*? (USE SHOWCARD)	Yes 1<br>No 2 *If no, go to T15*	T12
32	Do you **currently use smokeless tobacco** products **daily?**	Yes 1<br>No 2 *If no, go to T14aw*	T13
33	On average, how many **times a day/ week** do you use ….<br>(IF LESS THAN DAILY, RECORD WEEKLY)<br>(RECORD FOR EACH TYPE, USE SHOWCARD)<br>*Don't know 7777*	DAILY↓ WEEKLY↓	
		Snuff, by mouth └─┴─┴─┴─┘ └─┴─┴─┴─┘	T14a/T14aw
		Snuff, by nose └─┴─┴─┴─┘ └─┴─┴─┴─┘	T14b/T14bw
		Chewing tobacco └─┴─┴─┴─┘ └─┴─┴─┴─┘	T14c/T14cw
		Betel └─┴─┴─┴─┘ └─┴─┴─┴─┘	T14d/ T14dw
		Other └─┴─┴─┴─┘ └─┴─┴─┴─┘<br>*If other, go to T14other, if T13=No, go to T16, else go to T17*	T14e/T14ew
		Other (please specify): └─┴─┴─┴─┴─┴─┘<br>*If T13=No, go to T16, else go to T17*	T14other/ T14 otherw
34	In the **past**, did you **ever use** smoke-less tobacco products such as *[snuff, chewing tobacco, nasal snuff, khaini, surti, gutka]*?	Yes 1<br>No 2 *If no, go to T17*	T15
35	In the **past**, did you **ever use** smoke-less tobacco products such as *[snuff, chewing tobacco, nasal snuff, khaini, surti, gutka]* **daily**?	Yes 1<br>No 2	T16
36	During the past 7 days, on how many days did someone **in your home** smoke when you were present?	Number of days └─┴─┘<br>Don't know 77	T17
37	During the past 7 days, on how many days did someone smoke in closed areas **in your workplace** (in the build-ing, in a work area or a specific office) when you were present?	Number of days └─┴─┘<br>Don't know or don't work in a closed area 77	T18

Alcohol consumption			
The next questions ask about the consumption of alcohol.			
**Question**		**Response**	**Code**
38	Have you **ever** consumed an alcoholic drink such as beer, wine, spirits, fermented cider or *[jaad, raksi, tungba*? (USE SHOWCARD)	Yes 1<br>No 2 *If no, go to D1*	A1a
39	Have you consumed an alcoholic drink within the **past 12 months**?	Yes 1<br>No 2 *If no, go to D1*	A1b
40	During the past 12 months, **how frequently** have you had at least one alcoholic drink?<br><br>(READ RESPONSES, USE SHOWCARD)	Daily 1<br>5–6 days per week 2<br>1–4 days per week 3<br>1–3 days per month 4<br>Less than once a month 5	A2
41	Have you consumed an alcoholic drink within the **past 30 days**?	Yes 1<br>No 2 *If no, go to D1*	A3
42	During the past 30 days, on how many **occasions** did you have at least one alcoholic drink?	Number └─┴─┘<br>Don't know 77	A4
43	During the past 30 days, when you drank alcohol, **on average**, how many **standard alcoholic drinks** did you have during one drinking occasion?<br><br>(USE SHOWCARD)	Number └─┴─┘<br>Don't know 77	A5
44	During the past 30 days, what was the **largest number** of standard alcoholic drinks you had on a single occasion, counting all types of alcoholic drinks together?	Largest number └─┴─┘<br>Don't Know 77	A6
45	During the past 30 days, how many times did you have for **men**: **five or more** for **women**: **four or more** standard alcoholic drinks in a single drinking occasion?	Number of times └─┴─┘<br>Don't know 77	A7
46	During the past 30 days, when you consumed an alcoholic drink, how often was it with meals? Please do not count snacks.	Usually with meals 1<br>Sometimes with meals 2<br>Rarely with meals 3<br>Never with meals 4	A8
47	During each of the **past 7 days**, how many standard alcoholic drinks did you have each day?<br><br>(USE SHOWCARD)<br><br>*Don't know 77*	Monday └─┴─┘<br>Tuesday └─┴─┘<br>Wednesday └─┴─┘<br>Thursday └─┴─┘<br>Friday └─┴─┘<br>Saturday └─┴─┘<br>Sunday └─┴─┘	A9a<br>A9b<br>A9c<br>A9d<br>A9e<br>A9f<br>A9g

Diet			
The next questions ask about the fruit and vegetables that you usually eat. I have a nutrition card here that shows you some examples of local fruit and vegetables. Each picture represents the size of a serving. As you answer these questions please think of a typical week in the last year.			
**Question**		**Response**	**Code**
48	In a typical week, on how many days do you **eat fruit**?<br>*(USE SHOWCARD)*	Number of days └─┴─┘ If Zero days, go to D3<br>Don't know 77	D1
49	How many **servings** of fruit do you eat on **one** of those days? (USE SHOWCARD)	Number of servings └─┴─┘<br>Don't know 77	D2
50	In a typical week, on how many days do you **eat vegetables**? (USE SHOWCARD)	Number of days └─┴─┘ If Zero days, go to D5<br>Don't know 77	D3
51	How many **servings** of vegetables do you eat on one of those days? *(USE SHOWCARD)*	Number of servings └─┴─┘<br>Don't know 77	D4
52	What type of oil or fat is most often used for meal preparation in your household?<br>(USE SHOWCARD)<br>(SELECT ONLY ONE)	Mustard oil 1<br>Refined vegetable oil 2<br>Lard or suet 3<br>Butter or ghee 4<br>Noodles oil 5<br>Other 6 If other, go to D5 other<br>None in particular 7<br>None used 8<br>Don't know 77	D5
		Other (Please Specify)	D5 other
53	On average, how many meals per week do you eat that were not prepared at a home? By meal, I mean breakfast, lunch and dinner.	Number └─┴─┘<br>Don't know 77	D6
54	How much of the oil/ghee identified in D5 does your household consume?(Fill only one option)<br>1 deuwa (1 chauthai) = 125ml; 5 muthi = 250ml,<br>1 mana = 500ml 1 litre = 1000 ml	millilitres in a day └─┴─┴─┴─┘<br>millilitres in a week └─┴─┴─┴─┘<br>millilitres in a month └─┴─┴─┴─┘<br>Don't Know 77	X1
55	How many people of following age groups live in your household?<br>(Record for all the options applicable)	Less than 3 years └─┴─┘	X2a
		3 to 5 year └─┴─┘	X2b
		5 to 7 year └─┴─┘	X2c
		7 to 9 years └─┴─┘	X2d
		9 to 12 years └─┴─┘	X2e
		12 to 21 years └─┴─┘	X2f
		More than 21 years └─┴─┘	X2g

Dietary salt				
The next questions ask about your knowledge, attitudes and behaviour towards dietary salt. Dietary salt includes ordinary table salt, unrefined salt such as sea salt, iodised salt and salty sauces such as soya sauce or fish sauce. The following questions are on adding salt to food right before you eat it, how food is prepared in your home, eating processed foods that are high in salt such as chau chau, Lays chips, Kurkure, salty biscuits, canned fish, dry meat, titaura, preserved pickle, bhujia, mixtures, papad etc. and on controlling your salt intake. Please answer the questions even if you consider yourself to eat a diet low in salt.				
**Question**		**Response**		Code
56	How often do you **add salt** to your food before you eat it or as you are eating it?<br><br>(SELECT ONLY ONE)	Always<br>Often<br>Sometimes<br>Rarely<br>Never<br>Don't know	1<br>2<br>3<br>4<br>5<br>77	DS1
57	How often is **salt added** in cooking or preparing foods in your household?	Always<br>Often<br>Sometimes<br>Rarely<br>Never<br>Don't know	1<br>2<br>3<br>4<br>5<br>77	DS2
58	How often do you eat **processed food high in salt**, such as *chau chau, Lays, Kurkure, salty biscuits, canned fish, dry meat, titaura, preserved pickle, bhujia, mixtures, papad etc.*?<br><br>*(USE SHOWCARD)*	Always<br>Often<br>Sometimes<br>Rarely<br>Never<br>Don't know	1<br>2<br>3<br>4<br>5<br>77	DS3
59	**How much salt** do you think you consume?	Far too much<br>Too much<br>Just the right amount<br>Too little<br>Far too little<br>Don't know	1<br>2<br>3<br>4<br>5<br>77	DS4
60	Do you think that too much salt in your diet could cause a serious **health problem**?	Yes<br>No<br>Don't know	1<br>2<br>77	DS5
61	How important to you is **lowering the salt** in your diet?	Very important<br>Somewhat important<br>Not at all important<br>Don't know	1<br>2<br>3<br>77	DS6

Dietary salt continued			
**Question**		**Response**	Code
62	Do you do any of the following on a regular basis to **control your salt intake**? (RECORD FOR EACH)		
	Avoid/minimise consumption of processed foods	Yes 1<br>No 2	DS7a
	Look at the salt or sodium labels on food	Yes 1<br>No 2	DS7b
	Eat meals without adding salt at the table	Yes 1<br>No 2	DS7c
	Buy low salt/sodium alternatives	Yes 1<br>No 2	DS7d
	Cook meals without adding salt	Yes 1<br>No 2	DS7e
	Use spices other than salt when cooking	Yes 1<br>No 2	DS7f
	Avoid eating out	Yes 1<br>No 2	DS7g
	Other	Yes 1 *If Yes, go to DS7other*<br>No 2	DS7h
	Other (please specify)	└─┴─┴─┴─┴─┴─┴─┘	DS7other
63	Which type of salt do you use?	Crystal Salt 1<br>Powdered Salt without logo 2<br>Powdered salt with two children logo 3<br>Others 4 (If others go to X3 other<br>Others (Please Specify) └─┴─┴─┴─┴─┘	X3<br>X3 Other
64	How much salt does your family consume?(Fill only one option)<br>1 pathi crystal salt = 3,000 mg<br>1 mana crystal salt = 375 mg<br>1packet powdered salt = 1,000 mg	milligrams in a day └─┴─┴─┴─┘<br>milligrams in a week └─┴─┴─┴─┘<br>milligrams in a month └─┴─┴─┴─┘<br>Don't know 77	X4

Oral health				
The next questions ask about your oral health status and related behaviours.				
**Question**		**Response**		**Code**
65	How many **natural teeth** do you have?	No natural teeth<br>1 to 9 teeth<br>10 to 19 teeth<br>20 teeth or more<br>Don't know	1 *If no natural teeth, go to O4*<br>2<br>3<br>4<br>77	O1
**66**	How would you describe the **state of your teeth?**	Excellent<br>Very good<br>Good<br>Average<br>Poor<br>Very poor<br>Don't know	1<br>2<br>3<br>4<br>5<br>6<br>77	O2
**67**	How would you describe the **state of your gums?**	Excellent<br>Very good<br>Good<br>Average<br>Poor<br>Very poor<br>Don't know	1<br>2<br>3<br>4<br>5<br>6<br>77	O3
68	Do you have any **removable dentures**?	Yes<br>No	1<br>2 *If no, go to O6*	O4
69	Which of the following removable dentures do you have?<br>(RECORD FOR EACH)			
	An upper jaw denture	Yes<br>No	1<br>2	O5a
	A lower jaw denture	Yes<br>No	1<br>2	O5b
70	During the past 12 months, did your teeth or mouth cause any **pain or discomfort**?	Yes<br>No	1<br>2	O6
71	How long has it been since you last **saw a dentist**?	Less than 6 months<br>6–12 months<br>More than 1 year, but less than 2 years<br>2 or more years, but less than 5 years<br>5 or more years<br>Never received dental care	1<br>2<br>3<br>4<br>5<br>6 *If never, go to O9*	O7

72	What was the **main reason for your last visit** to the dentist?	Consultation/advice	*1*	O8
		Pain or trouble with teeth, gums or mouth	*2*	
		Treatment / Follow-up treatment	*3*	
		Routine check-up treatment	*4*	
		Other	5 *If other, go to O8other*	
		Other (please specify)	└─┴─┴─┴─┴─┘	O8 other
73	How **often do you clean** your teeth?	Never	1 *If Never, go to O13a*	O9
		Once a month	2	
		2–3 times a month	3	
		Once a week	4	
		2–6 times a week	5	
		Once a day	6	
		Twice or more a day	7	

Oral health continued			
**Question**		**Response**	**Code**
74	Do you use **toothpaste** to clean your teeth?	Yes 1<br>No 2 *If no, go to O12a*	O10
75	Do you use **toothpaste** containing **fluoride**?	Yes 1<br>No 2<br>Don't know 77	O11
76	Do you use any of the following to **clean your teeth**?<br>*(RECORD FOR EACH)*		
	Toothbrush	Yes 1<br>No 2	O12a
	Wooden toothpick	Yes 1<br>No 2	O12b
	Plastic toothpick	Yes 1<br>No 2	O12c
	Thread (dental floss)	Yes 1<br>No 2	O12d
	Charcoal	Yes 1<br>No 2	O12e
	Chewstick/miswak	Yes 1<br>No 2	O12f
	Other	Yes 1 *If Yes, go to O12other*<br>No 2	O12g
	Other (please specify)	└─┴─┴─┴─┴─┴─┴─┘	O12 other
77	Have you **experienced any of the following problems** during the past 12 months because of the **state of your teeth**? (RECORD FOR EACH)		
	Difficulty in chewing foods	Yes 1<br>No 2	O13a
	Difficulty with speech/trouble pronouncing words	Yes 1<br>No 2	O13b
	Felt tense because of problems with teeth or mouth	Yes 1<br>No 2	O13c
	Embarrassed about appearance of teeth	Yes 1<br>No 2	O13d

	Avoided smiling because of teeth	Yes 1<br>No 2	O13e
	Sleep is often interrupted	Yes 1<br>No 2	O13f
	Days not at work because of teeth or mouth	Yes 1<br>No 2	O13g
	Difficulty doing usual activities	Yes 1<br>No 2	O13h
	Less tolerant of spouse or people close to you	Yes 1<br>No 2	O13i
	Reduced participation in social activities	Yes 1<br>No 2	O13j
78	Are you currently suffering from dental caries?	Yes 1<br>No 2	014

Physical activity

Next I am going to ask you about the time you spend doing different types of physical activity in a typical week. Please answer these questions even if you do not consider yourself to be a physically active person. Think first about the time you spend doing work. Think of work as the things that you have to do such as paid or unpaid work, study or training, household chores, harvesting food and crops, fishing or hunting for food, seeking employment, walking uphill or downhill for routine work. In answering the following questions 'vigorous-intensity activities' are activities that require hard physical effort and cause large increases in breathing or heart rate, 'moderate-intensity activities' are activities that require moderate physical effort and cause small increases in breathing or heart rate.

	Question	Response	Code
**Work**			
79	Does your work involve vigorous-intensity activity that causes large increases in breathing or heart rate *[carrying or lifting heavy loads, digging or construction work]* for at least 10 minutes continuously? (USE SHOWCARD)	Yes 1<br>No 2 *If no, go to P4*	P1
80	In a typical week, on how many days do you do vigorous-intensity activities as part of your work?	Number of days └─┘	P2
81	How much time do you spend doing vigorous-intensity activities at work on a typical day?	Hours: minutes └─┴─┘ : └─┴─┘<br>hrs mins	P3 (a-b)
82	Does your work involve moderate-intensity activity that causes small increases in breathing or heart rate *[brisk walking, carrying light loads, manual washing clothes, mopping of floor, gardening at home]* for at least 10 minutes continuously?<br>*[INSERT EXAMPLES] (USE SHOWCARD)*	Yes 1<br>No 2 *If no, go to P 7*	P4
83	In a typical week, on how many days do you do moderate-intensity activities as part of your work?	Number of days └─┘	P5
84	How much time do you spend doing moderate-intensity activities at work on a typical day?	Hours: minutes └─┴─┘ : └─┴─┘<br>hrs mins	P6 (a-b)
**Travel to and from places**			
	The next questions exclude the physical activities at work that you have already mentioned. Now I would like to ask you about the usual way you travel to and from places. For example to work, for shopping, to market, to place of worship.		
85	Do you walk or use a bicycle *(pedal cycle)* for at least 10 minutes continuously to get to and from places?	Yes 1<br>No 2 *If no, go to P 10*	P7
86	In a typical week, on how many days do you walk or bicycle for at least 10 minutes continuously to get to and from places?	Number of days └─┘	P8
87	How much time do you spend walking or bicycling for travel on a typical day?	Hours: minutes └─┴─┘ : └─┴─┘<br>hrs mins	P9 (a-b)

Physical activity continued			
**Question**		**Response**	**Code**
**Recreational activity**			
The next questions exclude the work and transport activities that you have already mentioned. Now I would like to ask you about sports, fitness and recreational activities (leisure) like cycling, swimming, volleyball, badminton, yoga.			
88	Do you do any vigorous-intensity sports, fitness or recreational (leisure) activities that cause large increases in breathing or heart rate *[running or football]* for at least 10 minutes continuously?<br><br>(USE SHOWCARD)	Yes 1<br><br>No 2 *If no, go to P 13*	P10
89	In a typical week, on how many days do you do vigorous-intensity sports, fitness or recreational (leisure) activities?	Number of days └─┘	P11
90	How much time do you spend doing vigorous-intensity sports, fitness or recreational activities on a typical day?	Hours: minutes └─┴─┘ : └─┴─┘<br>hrs mins	P12<br><br>(a-b)
91	Do you do any moderate-intensity sports, fitness or recreational (leisure) activities that cause a small increase in breathing or heart rate *[brisk walking, cycling, swimming, volleyball, badminton, yoga]* for at least 10 minutes continuously?<br><br>*[INSERT EXAMPLES] (USE SHOWCARD)*	Yes 1<br><br>No 2 *If no, go to P16*	P13
92	In a typical week, on how many days do you do moderate-intensity sports, fitness or recreational (leisure) activities?	Number of days └─┘	P14
93	How much time do you spend doing moderate-intensity sports, fitness or recreational (leisure) activities on a typical day?	Hours: minutes └─┴─┘ : └─┴─┘<br>hrs mins	P15<br>(a-b)

Sedentary behaviour				
The following question is about sitting or reclining at work, at home, getting to and from places, or with friends including time spent sitting at a desk, sitting with friends, travelling in car or bus, reading, playing cards or watching television, but does not include time spent sleeping.<br><br>[INSERT EXAMPLES] (USE SHOWCARD)				
94	How much time do you usually spend sitting or reclining on a typical day?	Hours: minutes	└─┴─┘ : └─┴─┘<br>hrs mins	P16<br><br>(a-b)

Housing and energy (Indoor air pollution)				
The next questions ask about housing and energy.				
**Question**		**Response**		**Code**
95	Observe the roof material of house<br><br>(Don't ask the participants, just observe yourself)	Grass/leaves/reeds/thatch/wood/ mud/bamboo or mixed	1	X5
		Stone	2	
		Concrete	3	
		Tiles, slate, shingles	4	
		Bricks, stones and lime	5	
		Corrugated iron, zinc or other metal sheets	6	
		Others	7 (If others go to X5 other)	
		Others (Please specify)	└─┴─┴─┴─┴─┘	X5 Other
96	Observe the wall materials of house	Grass/leaves/reeds/bamboos /thatch or mixed	1	X6
		Mud/dirt	2	
		Unfired bricks	3	
		Wood	4	
		Fired bricks	5	
		Stone	6	
		Cement concrete	7	
		Others	8 (If others go to X6 other)	
		Others (please specify)	└─┴─┴─┴─┴─┘	X6 Other
97	Observe the floor materials of house	Mud/dirt	1	X7
		Wood/planks	2	
		Bamboo or logs	3	
		Cement	4	
		Bricks, stones and lime	5	
		Others	6 (If others go to X7 other)	
		Others (please specify)	└─┴─┴─┴─┴─┘	X7 Other
98	Do you have a separate room that is used as a kitchen?	Yes	1	X8
		No	2	
99	What is the main fuel for cooking in your house?	Wood/timber	1	X9
		Kerosene	2	
		LPG	3	
		Cow dung	4	
		Bio-gas	5	
		Straw and thatch	6	
		Others	9 (If others go to X9 other)	
		Others (please specify)	└─┴─┴─┴─┴─┘	X9 Other

Housing and energy continued				
Question		Response		Code
100	What type of stove do you use in house	Open fire	1	X10
		Mud stove	2	
		Smokeless stove	3	
		Kerosene stove	4	
		Gas stove	5	
		Others	6 (If others go to X10 other)	
		Others (please specify)	|__|__|__|_|__|	X10 Other
101	What is the main source of lighting for your house?	Kerosene	1	X11
		Pine wood fuel	2	
		Solar	3	
		Candle	4	
		Electricity	5	
		Others	6 (If others go to X11 other)	
		Others (please specify)	|__|__|__|_|__|	X11 Other

History of raised blood pressure				
Question		Response		Code
102	Have you ever had your blood pressure measured by a doctor or other health worker?	Yes 1<br>No 2	If no, go to H6	H1
103	Have you ever been told by a doctor or other health worker that you have raised blood pressure or hypertension?	Yes 1<br>No 2	If no, go to H6	H2a
104	Have you been told in the past 12 months?	Yes 1<br>No 2		H2b

105	Are you currently receiving any of the following treatments/advice for high blood pressure prescribed by a doctor or other health worker?		
	Drugs (medication) that you have taken in the past two weeks	Yes 1<br>No 2	H3a
	Advice to reduce salt intake	Yes 1<br>No 2	H3b
	Advice or treatment to lose weight	Yes 1<br>No 2	H3c
	Advice or treatment to stop smoking	Yes 1<br>No 2	H3d
	Advice to start or do more exercise	Yes 1<br>No 2	H3e
106	Have you ever seen a traditional healer for raised blood pressure or hypertension?	Yes 1<br>No 2	H4
107	Are you currently taking any herbal or traditional remedy for your raised blood pressure?	Yes 1<br>No 2	H5

History of diabetes			
**Question**		**Response**	**Code**
108	Have you ever had your blood glucose measured by a doctor or other health worker?	Yes 1<br>No 2 *If no, go to M1*	H6
109	Have you ever been told by a doctor or other health worker that you have raised blood glucose or diabetes?	Yes 1<br>No 2 *If no, go to M1*	H7a
110	Have you been told in the past 12 months?	Yes 1<br>No 2	H7b

111	Are you currently receiving any of the following treatments/advice for diabetes prescribed by a doctor or other health worker?		
	Insulin	Yes 1<br>No 2	H8a
	Drugs (medication) that you have taken in the past two weeks	Yes 1<br>No 2	H8b
	Special prescribed diet	Yes 1<br>No 2	H8c
	Advice or treatment to lose weight	Yes 1<br>No 2	H8d
	Advice or treatment to stop smoking	Yes 1<br>No 2	H8e
	Advice to start or do more exercise	Yes 1<br>No 2	H8f
112	Have you ever seen a traditional healer for diabe-tes or raised blood glucose?	Yes 1<br>No 2	H9
113	Are you currently taking any herbal or traditional remedy for your diabetes?	Yes 1<br>No 2	H10

STEP II. Physical measurements

CORE: Height and weight			
**Question**		**Response**	**Code**
114	Interviewer ID	└─┴─┴─┘	M1
115	Device IDs for height and weight	Height └─┴─┘<br>Weight └─┴─┘	M2a<br>M2b
116	Height	in centimetres (cm) └─┴─┴─┘.└─┘	M3
117	Weight<br>*If too large for scale 666.6*	in kilograms (kg) └─┴─┴─┘.└─┘	M4
118	**For women:** Are you pregnant?	Yes 1 *If yes, go to M 8*<br>No 2	M5
**CORE: Waist**			
119	Device ID for waist	└─┴─┘	M6
120	Waist circumference	in Centimetres (cm) └─┴─┴─┘.└─┘	M7
**CORE: Blood pressure**			
121	Interviewer ID	└─┴─┴─┘	M8
122	Device ID for blood pressure	└─┴─┘	M9
123	Cuff size used	Small 1<br>Medium 2<br>Large 3	M10
124	Reading 1	Systolic (mmHg) └─┴─┴─┘<br>Diastolic (mmHg) └─┴─┴─┘	M11a<br>M11b
125	Reading 2	Systolic (mmHg) └─┴─┴─┘<br>Diastolic (mmHg) └─┴─┴─┘	M12a<br>M12b
126	Reading 3	Systolic (mmHg) └─┴─┴─┘<br>Diastolic (mmHg) └─┴─┴─┘	M13a<br>M13b
127	During the past two weeks, have you been treated for raised blood pressure with drugs (medication) prescribed by a doctor or other health worker?	Yes 1<br>No 2	M14

Hip circumference and heart rate				
128	Hip circumference	in centimetres (cm)	└─┴─┴─┘.└─┘	M15
129	Heart rate			
	Reading 1	Beats per minute	└─┴─┴─┘	M16a
	Reading 2	Beats per minute	└─┴─┴─┘	M16b
	Reading 3	Beats per minute	└─┴─┴─┘	M16c

STEP III Biochemical measurements

Blood glucose			
**Question**		**Response**	**Code**
130	During the past 12 hours have you had anything to eat or drink, other than water?	Yes 1<br>No 2	B1
131	Technician ID	└─┴─┴─┘	B2
132	Device ID	└─┴─┘	B3
133	Time of day blood specimen taken (24 hour clock)	Hours: minutes └─┴─┘ : └─┴─┘<br>hrs mins	B4
134	Fasting blood glucose	mg/dl └─┴─┴─┘ . └─┘	B5
135	Today, have you taken insulin or other drugs (medication) that have been prescribed by a doctor or other health worker for raised blood glucose?	Yes 1<br>No 2	B6
**Blood lipids**			
136	Device ID	└─┴─┘	B7
137	Total cholesterol	mg/dl └─┴─┴─┘ . └─┘	B8
138	During the past two weeks, have you been treated for raised cholesterol with drugs (medication) prescribed by a doctor or other health worker?	Yes 1<br>No 2	B9
**Triglycerides and HDL cholesterol**			
139	Triglycerides	mg/dl └─┴─┴─┘ . └─┘	B10
140	HDL Cholesterol	mg/dl └─┴─┴─┘ . └─┘	B11
